# Improving paediatric and neonatal care in rural district hospitals in the highlands of Papua New Guinea: a quality improvement approach

**DOI:** 10.1179/2046905513Y.0000000081

**Published:** 2014-05

**Authors:** Martin Sa’avu, Trevor Duke, Sens Matai

**Affiliations:** 1 Mendi Hospital, Southern Highlands Province, Papua New Guinea; 2 Centre for International Child Health, University of Melbourne, and School of Medicine and Health Sciences, University of PNG; 3 Biomedical Engineer, Pacific Technical Assistance Mechanism

**Keywords:** Quality of paediatric care, Neonatal care, Oxygen therapy, District hospitals, Oxygen concentrators, Rural health

## Abstract

**Background:**

In developing countries such as Papua New Guinea (PNG), district hospitals play a vital role in clinical care, training health-care workers, implementing immunization and other public health programmes and providing necessary data on disease burdens and outcomes. Pneumonia and neonatal conditions are a major cause of child admission and death in hospitals throughout PNG. Oxygen therapy is an essential component of the management of pneumonia and neonatal conditions, but facilities for oxygen and care of the sick newborn are often inadequate, especially in district hospitals. Improving this area may be a vehicle for improving overall quality of care.

**Method:**

A qualitative study of five rural district hospitals in the highlands provinces of Papua New Guinea was undertaken. A structured survey instrument was used by a paediatrician and a biomedical technician to assess the quality of paediatric care, the case-mix and outcomes, resources for delivery of good-quality care for children with pneumonia and neonatal illnesses, existing oxygen systems and equipment, drugs and consumables, infection-control facilities and the reliability of the electricity supply to each hospital. A floor plan was drawn up for the installation of the oxygen concentrators and a plan for improving care of sick neonates, and a process of addressing other priorities was begun.

**Results:**

In remote parts of PNG, many district hospitals are run by under-resourced non-government organizations. Most hospitals had general wards in which both adults and children were managed together. Paediatric case-loads ranged between 232 and 840 patients per year with overall case-fatality rates (CFR) of 3–6% and up to 15% among sick neonates. Pneumonia accounts for 28–37% of admissions with a CFR of up to 8%. There were no supervisory visits by paediatricians, and little or no continuing professional development of staff. Essential drugs were mostly available, but basic equipment for the care of sick neonates was often absent or incomplete. Infection control measures were inadequate in most hospitals. Cylinders were the major source of oxygen for the district hospitals, and logistical problems and large indirect costs meant that oxygen was under-utilized. There were multiple electricity interruptions, but hospitals had back-up generators to enable the use of oxygen concentrators. After 6 months in each of the five hospitals, high-dependency care areas were planned, oxygen concentrators installed, staff trained in their use, and a plan was set out for improving neonatal care.

**Interpretation:**

If MGD-4 targets for child health are to be met, reducing neonatal mortality and deaths from pneumonia will have to include better quality services in district hospitals. Establishing better oxygen supplies with a systems approach can be a vehicle for addressing other areas of quality and safety in district hospitals.

## Introduction

District hospitals are central to community health and child survival.^1^,^2^ They play a vital role in providing first-level inpatient and outpatient care for patients who have been referred by primary care-providers. They play an important role in training health-care workers, implementing immunization and other public health programmes and providing necessary data to national health-planners. In resource-poor settings, district hospitals need to maintain a minimal standard of care and deliver essential and cost-effective services.^3^

In 2011, pneumonia took the lives of 1.3 million children in developing countries.^4^ In Papua New Guinea (PNG), pneumonia is the leading cause of childhood deaths. In ten provincial hospitals in 2010–11, pneumonia caused over 6900 admissions with 439 deaths, and case fatality rates were 5.6% in 2010 and 6.1% in 2011.

As neonatal mortality accounts for about 40% of child mortality globally, it is now well recognized that improving neonatal outcomes is essential for achieving MDG-4 targets.^4^,^5^ In ten provincial hospitals in PNG in 2010 and 2011, neonatal conditions accounted for 4500 admissions and 468 deaths with case fatality rates of 9.4 and 10.3%, respectively.

Pneumonia and neonatal conditions together account for over 50% of hospital admissions and deaths. The most serious complication of pneumonia is hypoxaemia (low levels of oxygen in the blood), and this is also common in many neonatal conditions. The risk of death from pneumonia or neonatal conditions is increased many times in the presence of hypoxaemia.^6^

Besides antibiotics to treat pneumonia and neonatal sepsis, WHO recommends that children with very severe pneumonia and neonatal conditions that result in hypoxaemia be given oxygen therapy.^7^–^9^ Because of transport difficulties, oxygen is often unavailable in remote district hospitals.^10^–^13^

Improving the reliability of oxygen systems using oxygen concentrators and hypoxaemia detection using pulse oximetry has been shown to reduce case fatality by up to 35% in treating children with severe pneumonia in provincial hospitals in PNG.^14^ Expansion of this programme into the rural district hospitals could reduce deaths from pneumonia and be a vehicle for improving quality of care in district hospitals. However, such technology needs to be supported by systems approaches and reliable power supplies. Experience suggests that power disruptions in rural hospitals are frequent, but this problem has never been quantified, so it has not been possible to know if such equipment is appropriate to these environments.

We sought to better understand the quality of care provided for children in district hospitals in the highlands of PNG, including neonatal services, the resources available for oxygen administration, and the infrastructure to support them. This formed the basis of practical interventions to improve quality of paediatric and neonatal care.

## Methods

In 2010 and 2011, five rural district hospitals in the highlands of PNG were assessed and information on the following was systematically gathered:

- Resources available in the paediatric units, including wards, facilities, equipment and staffing;
- Clinical data on overall admissions and deaths, pneumonia admissions and deaths and neonatal admissions and deaths during the previous 3 years;
- Existing sources of oxygen and oxygen supplies;
- Electricity supply and its reliability.

Data were collected using structured survey forms for each of the above categories. More details are provided below.

### Hospital resources and staffing

Where they existed, the children’s wards and neonatal wards were evaluated, recording bed capacity, available diagnostic equipment and laboratory facilities, supplies of essential medicines, and facilities to maintain hygiene and infection control. Data on medical and nursing staff were also recorded, including their training and any continuing professional development in paediatrics and neonatal care.

### Clinical data-collection

Records of patient information, demographic data, diagnosis and outcome are kept on the wards in the admission books. Clinical outcome data for up to 43 months, from January 2007 to July 2010, were retrieved from the ward admission books and hospital records. All paediatric admissions and deaths, pneumonia admissions and deaths, and neonatal admissions and deaths were recorded. From these data, case-fatality rates were calculated. Where hospitals had incomplete data, further information was sought from the monthly reports from the nursing manager and from the provincial health office. Median annual admissions, deaths and case fatality rates are presented.

### Oxygen supplies and oximetry

The number of cylinders available was recorded, their volume capacity established, and the hospitals’ oxygen procurement records checked. A record was made of any oxygen concentrators and pulse oximeters that were available, how the equipment was obtained, whether it was functioning, its clinical use, and whether any servicing had been carried out. Oxygen costs for each hospital were calculated.

### Reliability of electricity supply

Since oxygen concentrators depend on the continuous supply of electricity, the reliability of the electricity supply to the five hospitals was assessed using a Grabinter LS164/IP65 electrocorder (Acksen, UK, www.electrocorder.com). These have an input supply of a 50–300-volt alternating current and 50–60 Hz at 0.1A maximum. Electrocorders were plugged into a mains electrical socket in each hospital. Over 4–5 weeks, data on the number and duration of interruptions to the hospitals’ power supplies, whether these occurred during the day or at night-time, and the percentage of time for which the hospitals had power were recorded. Data from the electrocorders were downloaded for analysis using in-built software.

### Drawing a plan for proposed installation

Based on the above information, options for oxygen therapy were discussed. After discussion with each hospital, plans for installing oxygen concentrators were drawn up. With the help of the ward manager and the administrators, an appropriate location for a high-dependency area was identified on the children’s ward or in the section of the general ward allocated to children. The proposed installation site was created by the biomedical engineer who measured the space and drew a floor plan with dimensions showing total number of beds and the location of the electrical sockets for the concentrators. The total number of components, the length of oxygen tubing and electrical conduit and appropriate fittings were identified. A plan of the proposed installation was drawn for each hospital, based on their specific needs. In the subsequent 6 months, training of staff and installation of oxygen concentrators and pulse oximeters was undertaken in each hospital, and plans for improving neonatal care were discussed.

### Data analyses and ethical clearance

The data from this study were quantitative and qualitative. Data were entered into a Microsoft Excel spreadsheet using SPSS version 10, and descriptive analyses conducted. For the data on electrical status, software called Electro-soft for uploading and analysis was used.

Before the study commenced, ethical clearance was obtained from the University of Papua New Guinea Research Committee, and permission for data-gathering and collaboration was granted by each of the hospitals involved.

## Results

### Characteristics of the hospitals

Five district hospitals in the highlands provinces of PNG were involved in the survey. These were Ialibu, Kompiam, Mambisanda, Mingende and Paiam. Three hospitals are run by church organizations: Kompiam (Baptist), Mambisanda (Lutheran) and Mingende (Catholic). Ialibu Hospital is run by the provincial government and Paiam Hospital is run by a gold mining company under the Porgera Development Authority.

Only one district hospital had a children’s ward and a separate section for newborns. Two hospitals had a children’s ward but no neonatal ward, and two hospitals had a common ward for adults and children (Table 1).

**Table 1:** Paediatric services at the five district hospitals for the years 2007 to 2010

	Ialibu	Mingende	Mambisanda	Kompiam	Paiam
Children’s ward	✓	✓	✓	✗	✗
Neonatal wards	✗	✓	✗	✗	✓
Bed capacity in children’s ward	24	17	22	Common ward*	Common 8-bed ward*
Medical officers (MO)	0–1	0–1	2–3	2–3	3–4
Health Extension Officers (HEO)	0–1	0–3	0–1	0	0–2
Paediatric nurses	2	3	1–3	0	1
General Nurses (NO)	18	18–25	18–19	10	26
Community Health Workers (CHW)	12	18–28	23–26	11–12	16

* Adults and children.

Hospitals with no neonatal facility nursed all babies with their mothers. This is an excellent arrangement as it encourages breast-feeding and skin-to-skin contact to maintain the infant’s temperature, and is less likely to result in cross-infection. However, it may be sub-optimal for very sick newborns.

Some hospitals were over-crowded, and some appeared under-utilized. In Paiam, eight beds were allocated to children in the general ward, all of which were occupied at the time of assessment. Six other sick children were nursed on the floor in the corridor. Some others hospital wards had no children at the time of assessment.

### Human resources and continuing professional development

In all but one hospital there were trained paediatric nurses, but their numbers were few, not enabling one per shift in any hospital. Furthermore, most postgraduate-trained paediatric nurses had been given administrative duties and were not actively involved in the clinical care of children.

No nurses in the hospitals had attended any training in paediatrics or neonatal care in the previous 5 years, including no exposure to IMCI, despite it being a national programme for improving primary child care. There was little or no professional development for nurses, apart from HIV training in two hospitals in 2010. No paediatrician had visited any of the district hospitals in the previous 5 years.

### Facilities and services with a focus on neonatal care

Table 2 describes neonatal equipment and consumables. Most essential drugs were available in all district hospitals. There were few exceptions: antibiotic eye ointment and parenteral aminophylline in two and one hospital, respectively.

**Table 2:** Neonatal and paediatric equipment and facilities in the five district hospitals

	Ialibu	Mingende	Mambisanda	Kompiam	Paiam
**Neonatal ward layout & facilities**					
Separate neonatal ward	✗	✓	✗	✗	✓
Labour ward with clean bed and linen	✗	✓	✓	✗	✓
Cot/bed capacity	0	7	2	1	4
Fridge for medicine/vaccine, expressed breast milk	✓	✓	✓	✓	✓
Wall thermometer	✗	✗	✗	✗	✗
**Maternal & family facilities**					
24 hour maternal/baby access	✓	✓	✓	✓	✓
Rooming-in facilities	✗	✗	✗	✗	✗
**Infection control**					
Hand-washing facility	✗	✓	✓	✓	✓
Soap available	✗	✓	✓	✓	✗
Hand towels	✗	✓	✓	✗	✗
Safe sharps disposal	✓	✓	✓	✓	✓
Waste disposal	✓	✓	✓	✓	✓
Chlorhexidine/povidine iodine solution	✓	✓	✓	✓	✓
Cord clamps/gentian violet	✗	✓	✗	✗	✗
Detergent	✓	✓	✗	✓	✓
Infection control policy	✗	✓	✗	✗	✗
**Ward statistics**					
Admissions, discharge, death records available	✓	✓	✓	✓	✓
Regular ward mortality & morbidity meeting	✗	✗	✗	✗	✗
**Nursery equipment & supplies**					
Scales	✓	✓	✓	✓	✓
Thermometers	✓	✓	✓	✓	✓
Suction machine & accessories	✗	✓	✓	✓	✗
Radiant warmers/incubators	0	1	0	1	2
Emergency resuscitation area	✗	✓	✗	✓	✓
Neonatal resuscitation equipment	✗	✗	✗	✓	✗
Apnoea monitors	✗	✗	✗	✗	✗
Phototherapy system	✗	✗	✗	✗	✓
IV sets, burettes	✓	✓	✓	✓	✓
Syringes, needles, cannulas	✓	✓	✓	✓	✓
Sterile gloves & masks	✓	✓	✓	✓	✓
Glucometer & accessories	✗	✗	✗	✗	✗
Breast pump	✗	✓	✗	✗	✗
Baby feeding cups & spoons	✗	✓	✗	✗	✗
**Essential drugs & supply**					
Adrenaline	✓	✓	✓	✓	✓
Aminophylline/theophylline	✓	✓	✗	✓	✓
Ampicillin/amoxicillin injection	✓	✓	✓	✓	✓
Benzyl penicillin	✓	✓	✓	✓	✓
Ceftriaxone/cefotaxime	✓	✓	✓	✓	✓
Chloramphenicol injection/oral	✓	✓	✓	✓	✓
Cloxacillin injection	✓	✓	✓	✓	✓
Gentamicin injection	✓	✓	✓	✓	✓
Nystatin drops	✓	✓	✓	✓	✓
Phenobarbitone injection	✓	✓	✓	✓	✓
Tetracycline eye ointment/drops	✗	✓	✓	✗	✓
Vitamin K injection	✓	✓	✓	✓	✓
Vaccines (BCG, Hep B)	✓	✓	✓	✓	✓
Sterile water for injection	✓	✓	✓	✓	✓
IV fluids: 5%/10% dextrose, saline, Hartman’s solution	✓	✓	✓	✓	✓
Alcohol swabs	✓	✓	✓	✓	✓
**Laboratory & imaging facilities**					
FBC	✗	✓	✓	✓	✓
Blood smear for malaria	✓	✓	✓	✓	✓
Blood/CSF culture	✗	✗	✓	✗	✗
UEC	✗	✓	✗	✓	✓
Blood grouping & x-match	✗	✓	✓	✓	✓
LFT	✗	✓	✗	✓	✓
Bilirubin	✗	✓	✗	✗	✓
Urinalysis	✗	✓	✓	✓	✓
CSF protein/sugar	✗	✓	✓	✓	✗
X-rays	✓	✓	✓	✓	✓
Ultrasonography	✗	✗	✓	✗	✓

Basic neonatal equipment including room thermometers, suction machines, apnoea monitors, breast-pumps and glucometers were not available in some of the hospitals. Most hospitals had incomplete resuscitation equipment with important items missing, e.g. neonatal masks or inflation resuscitation bags. There were no trolleys to enable easy access to resuscitation drugs or equipment.

Although most wards had hand-washing facilities (a basin and running water from a tap), soap and hand-towels were not readily available in two and three hospitals, respectively. Infection control measures, policies and guidelines were displayed in only one hospital.

All hospitals could undertake plain radiographs. Bacteriological culture was possible in only one hospital. In the one government-run hospital, all laboratory facilities were run-down with no replacement, maintenance or commodities provided for more than 5 years. This hospital could only do a blood smear for malaria parasites.

Ward statistics on admissions, discharges, deaths, referrals and transfers were well kept in common record books. However, none of the hospitals had analyzed the records or held mortality and morbidity meetings in the previous 5 years.

### Clinical data

The median number of paediatric admissions in these district hospitals was between 232 and 840 patients per year (Table 3). Children and neonates with pneumonia accounted for 28–37% and 5–14% of paediatric admissions, respectively.

**Table 3:** Annual admissions, mortality and case fatality rates in children and neonates in the five district hospitals

	Ialibu	Mingende	Mambisanda	Kompiam	Paiam
**All children**					
Median annual admissions (IQR)	238 (210–246)	840 (505–924)	302 (169–306)	232 (142–244)	485 (389–532)
Median annual deaths (IQR)	7 (5–9)	24 (18–33)	12 (6–17)	7 (3–12)	29 (27–37)
Total CFR, %	2.9	2.8	3.9	3.0	5.9
Median annual pneumonia admissions (IQR)	83 (68–128)	310 (224–320)	85 (49–96)	65 (45–92)	167 (160–180)
Pneumonia admissions, % total admissions/year	35	37	28	28	34
Median deaths from pneumonia/year (IQR)	2 (0–3)	5 (3–7)	3 (1–7)	2 (1–5)	14 (12–16)
Pneumonia CFR (%)	2.4	1.6	3.5	3.1	8.4
**Neonates**					
Median annual admissions (IQR)	33 (27–44)	90 (68–102)	33 (20–40)	11 (4–16)	51 (38–65)
Median annual deaths (IQR)	0.5 (0–2)	11 (8–3)	3 (1–4)	1 (1–4)	8 (4–10)
Total CFR (%)	1.5	12.2	9	9	15.6

The overall case fatality rate (CFR) in children ranged from 3% to 6% and the CFR owing to pneumonia ranged from 1% to 8%. Ialibu hospital had a low neonatal CFR of 1.5%, while the CFR in the other hospitals ranged from 9% to 15%. In Ialibu there were very low admission numbers because of a lack of neonatal facilities.

Paiam had quite high overall case fatality rates of 6% in children overall, 8% in those with pneumonia and 15% in neonates. Compared with the other hospitals, Mingende had the highest admission rates, but their CFRs were lower than in the others (Table 3).

### Resources for oxygen and monitoring hypoxaemia

Cylinders were the main source of oxygen supply. Most of the oxygen was pre-paid by the National Department of Health through the Area Medical Store (AMS) in Mt Hagen, Western Highlands Province, and therefore direct costs were not borne by the district hospitals. However, indirect costs of transport were high, and, along with logistical problems, were often cited as reasons for frequent running out of oxygen. As most hospitals were not directly paying for oxygen, procurement records were not kept up-to-date at the hospitals. Therefore, records of costs and purchasing were obtained directly from a central location, the AMS in Mt Hagen. Cost analysis was carried out by obtaining the price list of each type of cylinder used and the total number of cylinders supplied to the respective hospitals during the same period. The direct oxygen costs over 3 years ranged from K1160 for eight cylinders in Paiam (which was also receiving oxygen cylinders from the Porgera mine) to K25,500 for 263 cylinders in Mingende (1 Kina  =  $US 0.44).

Besides lack of transport or funding for transport of oxygen, other commonly cited barriers included problems that occurred on the roads, including land-slips and tribal conflicts which limited access to oxygen cylinders from the AMS.

All hospitals had a pulse oximeter. However, they were available only in the operating theatres and were used by anaesthetic technical officers. Pulse oximeters were not used in the wards for monitoring sick patients. Paediatric sensor probes for the pulse oximeters were available in only two of the five hospitals, and no hospital had sensor probes to fit on neonates. Much of this equipment was donated and it was commonly stated that it was difficult to seek support for maintenance. Most hospitals did not have a biomedical engineer or technician.

### Reliability of electricity supplies

The hospitals’ power came from the following sources:

- Ialibu used the mains PNG power supply and a standby diesel generator with a manual control;
- Mingende used the mains PNG power supply and a standby diesel generator with an automatic switch;
- Mambisanda had its own hydro-electricity and a standby diesel generator with auto-switch;
- Paiam was using power from the Porgera gold mine and a standby diesel generator with automatic switch;
- Kompiam had solar power for lighting and light work during the day and a diesel generator at night and on special operational days when there was to be a heavy workload in the operating theatre or for maintenance and repairs.

Table 4 lists the main data from electrocorder recordings in each hospital. Power disruptions occurred several times a day in most hospitals. They were mostly of short duration (median 3 minutes), but sometimes lasted more than a day.

**Table 4:** Electrical status of each district hospital (voltage threshold at 220–240V

	Ialibu	Mingende	Mambisanda	Kompiam	Paiam
Power source	Main PNG power	Main PNG power	Local hydro	Local solar	Pogera mine
Standby diesel generator	✓	✓	✓	✓	✓
Data logging period, days	40	30	42	29	41
Power is on, % time	69	95	99	38	99
**Interruptions**					
Total number	187	881	119	1946	30
Average/week	33	205	20	470	5
Average/month	142	892	86	2042	22
Total day-time, 06:00–17:59 hrs	87	640	54	1903	21
Total night-time, 18:00–5:59 hrs	100	241	65	43	9
Average duration*	01:35:06	00:02:33	00:03:07	00:13:19	00:06:34
Maximum duration	12:49:08	06:05:19	01:29:05	20:09:32	03:08:30
Average time between	03:33:59	00:46:36	08:26:13	00:08:08	08:44:27

* Hours: minutes: seconds.

Three hospitals had power available for more than 90% of the time. Kompiam and Ialibu were exceptions: both hospitals had regular and prolonged interruption of electricity supplies. Kompiam Hospital had a diesel-powered generator that was selectively activated from 6 p.m. to 10 p.m. and during the day for special operations and other heavy work for which electricity was needed, such as hospital maintenance or building. Although this resulted in more power interruptions than in the other hospitals, according to hospital administrators there were good sources of back-up power which could be activated if necessary (including supplementary solar power), and which was available to run concentrators. Mingende, Paiam and Mambisanda Hospitals had automatic switches to activate their diesel generators immediately the main power supply went off, so these hospitals had only brief, albeit frequent power blackouts.

### Follow-up: practical interventions for quality improvement approach

The floor plan for high-dependency areas (HDU) where oxygen concentrators were to be placed was decided in discussion with the paediatric ward managers and administrators. Measurements were taken and a floor plan drawn up.

**Figure 1:**
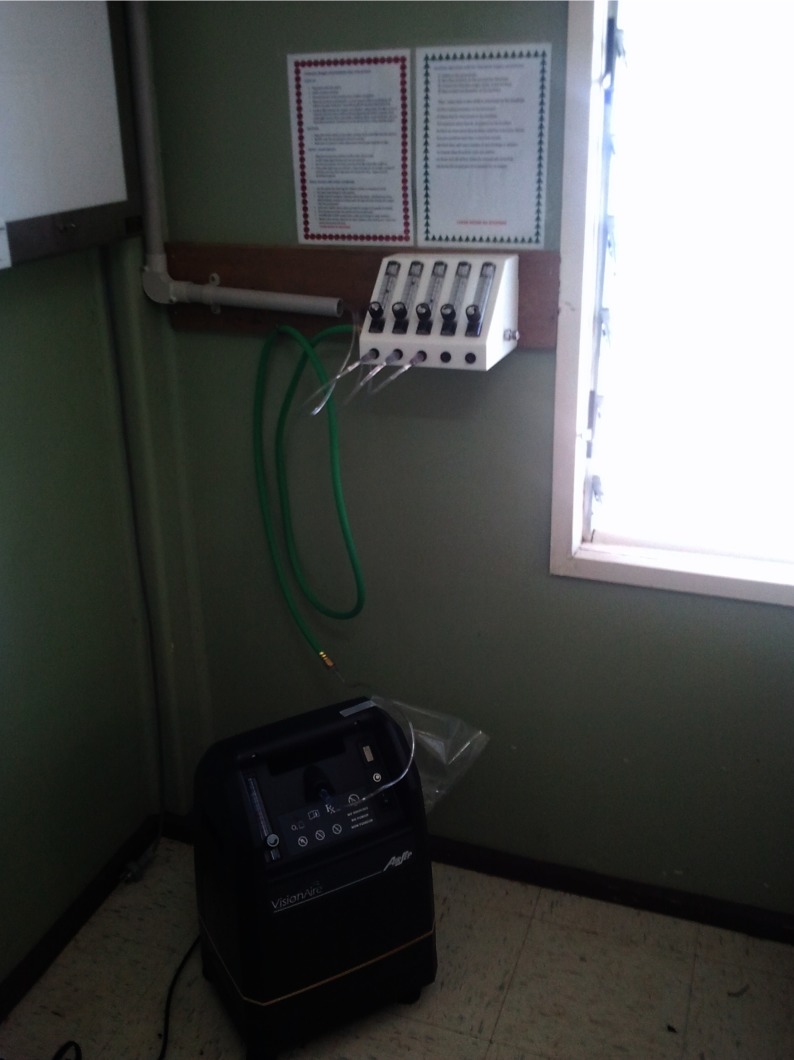
The oxygen concentrator after installation. Note the laminated instructions on the wall, the flow meter connected to the concentrator oxygen outlet, and the oxygen tubing running from each flow meter outlet through conduit to reach each bed-side

**Figure 2:**
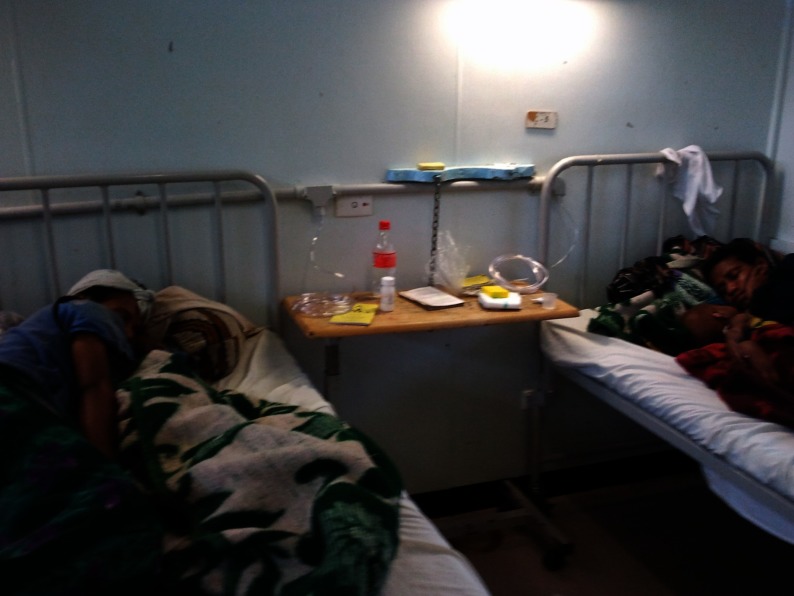
Practical demonstration of the use of oxygen concentrators by a nurse

Six months after the assessment was carried out, oxygen concentrators and pulse oximeters were installed in a return visit during which discussion of the other priorities in neonatal care and management of pneumonia commenced. Two 5-L/min oxygen concentrators (Visionairre, Airsep Corporation, New York) and a pulse oximeter (Bitmos) with a sensor probe suitable for children and one for neonates (CCyS, Madrid) were installed. The installation process was as follows:

- One concentrator was fixed to the nursing station in the paediatric ward or section of the general ward according to the ground plan initially drawn and the second was for use in any convenient location including the adult wards (Fig. 1);
- A Sure-flow meter (Airsep Corporation) was mounted to a wall near the nursing station. The Sure-flow meter is a multi-flow meter which distributes and controls independently the amount of oxygen flowing to individual patients;
- Tubing was connected from the oxygen concentrator to the flow meter mounted at the nursing station;
- Five pieces of oxygen tubing were connected to the respective bedsides with outlets to each bed (Fig. 2).

Depending on case-loads and the ward space, 3–5 beds were allocated for patients requiring oxygen in the HDU section, and installations were completed successfully.

Training was done both during the visit for baseline data collection and at the time of concentrator installation. Training involved hospital administrators, medical officers, health extension officers, ward managers and general nursing staff and, where possible, a technician in each hospital. This involved teaching on:

- The clinical burden of pneumonia and its complications;
- The importance of correctly identifying hypoxaemia, using oximetry and ready availability of oxygen;
- The role of oxygen in neonatal care;
- Distribution of the technical materials: the WHO Pocket Book of Hospital Care for Children and PNG Standard Treatment Books to doctors, health extension officers and nursing ward managers. Other resources used included the WHO Clinical Use of Oxygen manual;
- The importance of infection control measures;
- How to improve neonatal care.

**Figure 3:**
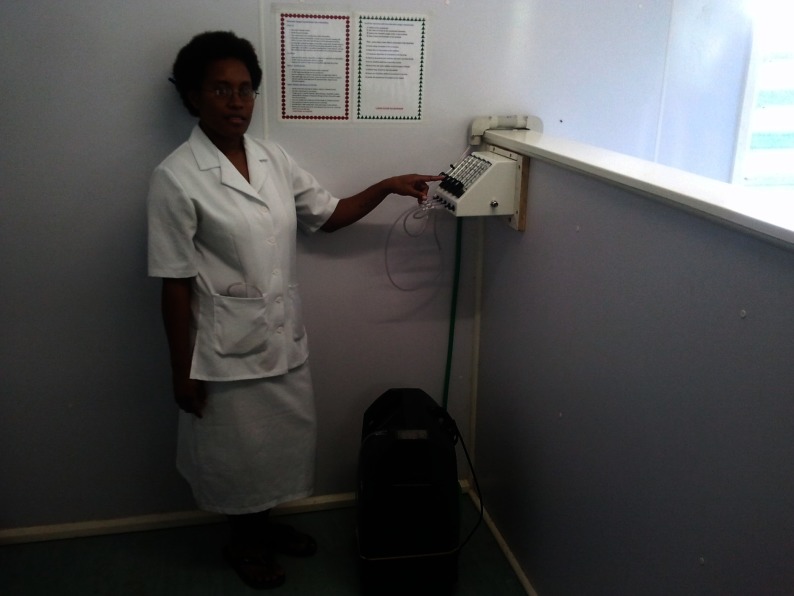
Oxygen outlets at the bedside of children and their mothers

Practical sessions with clinical case scenarios were arranged, including how to use clinical guidelines and oxygen equipment in everyday clinical care of newborns and children with pneumonia (Fig. 3).

Guidelines on the oxygen concentrators, pulse oximetry and clinical use of oxygen therapy were developed: when to give oxygen, how to give it, how much to give and when to wean children off oxygen. These guidelines were bound or laminated and placed on the walls of the high-dependency areas.

## Discussion

A step-by-step quality improvement approach can achieve progress in rural district hospitals to improve the management of common paediatric problems. Such an approach, which began with targeted collection of data on quality-of-care issues, with an initial practical intervention of improving oxygen systems, is described.

Other studies of quality improvement based in district hospitals in Kenya have focused on guidelines, training and supervision.^15^ These have been large projects, with an estimated cost of $3.6 million annually for scale-up in Kenya. The approach described in the present study is more modest, based on a local paediatrician being involved in supporting a quality improvement process in a series of district hospitals, and recognizing that in some hospitals infrastructure limitations are obstacles to quality.

Despite frequent interruptions to power supplies, it was thought in all hospitals that oxygen concentrators would be a better option than their current sources of oxygen. Cost comparisons between different sources of oxygen support these decisions.^16^ Lack of reliable oxygen supplies is just one gap identified in the care of neonates and children with pneumonia, but having a practical solution opened up discussion about taking steps to address other areas.

District hospitals function under extremes of social, environmental, geographic and financial conditions. They have limited manpower and resources, and poor logistic support, yet they commit themselves to maintain basic services to local communities. Many of the facilities for managing sick children in district hospitals in PNG have not been improved in the last decade, and certain facilities and services in some hospitals had not kept pace with community needs.

Many district hospitals have only a general ward that caters for both adults and children together. The problems of overcrowding, competition for space and cross-infection increase with a rising population. A separate paediatric ward, and a section of a ward with a neonatal unit, staffed by a paediatric nurse, is needed in a rural hospital.

Paediatric nurses have a large role to play in the management of children and neonates in district hospitals where the medical officer is often a general practitioner and busy with adult medical, obstetric or surgical problems.^17^ Why many paediatric nurses in the district hospitals have moved from active clinical care to administrative roles is not clear. It could be that too few have higher qualifications, so that those who do, even if the higher training is largely clinical, are preferred for administrative roles. If graduate paediatric nurses are not recognized and actively engaged in the role for which they are trained, it is easier for them to stream into administrative roles than to remain at large for general duties.

More paediatric nurses are needed. As has been achieved with midwifery, a long-term plan for paediatric training of nurses in district hospitals should be put in place, supported and assisted by the provincial government and health authorities. A regional course in the highlands of PNG with a population of over 2 million people can be easily justified.

No staff in the five district hospitals had been engaged in training or professional development in the past 5 years and no hospital had been visited by the provincial health authorities or paediatricians to support district paediatric services. Perception of neglect can have a negative effect on performance, morale and outcomes at district hospitals.

A quality improvement approach that encompasses assessment, problem identification, designing and implementing solutions can be effective. This process is not rapid, and it needs to be consistent and continuous to achieve sustained results. Such an approach should be coupled with continuing professional development of staff. Regular visits and assessment by the provincial health team and provincial paediatrician also have a positive effect on staff morale and stimulate competitive performance.

This process was commenced by reviewing facilities for neonatal care and for managing pneumonia, and addressing a major need for improved and more efficient oxygen systems. This allowed the identification of other problems which will be addressed. Solutions and resources are available for each of the key problems identified (Table 5).

**Table 5:** Gaps in services at the district hospitals, potential solutions and technical resources available

Priority item or gap in services	Intervention	Resource/programme
Lack of paediatric & neonatal areas in some hospitals	Establish dedicated areas for hospitalized children & a model of care for unwell newborns	
Oxygen therapy for pneumonia & neonatal care	Implementation of an oxygen system that includes concentrators & oximetersTraining in pneumonia management & use of oxygenEstablish high-dependency areas for the care of the sickest children	WHO Clinical Use of Oxygen http://video.rch.org.au/cich/The_Clinical_Use_of_Oxygen_November_2011.pdfWHO Hospital Care for Children and training CD, www.ichrc.org
Infection control	Improve soap or alcohol hand-wash	
Neonatal care	Improve basic neonatal equipmentTraining in care of the sick newbornwith sepsis or low birthweightNeonatal resuscitation training for birth asphyxia	Minimal standards of neonatal care^20^WHO Hospital Care for Childrenwww.ichrc.orgEssential Early Newborn Care https://apps.who.int/rht/documents/MSM96-13/essential_newborn_care.htmEmergency Obstetric Care http://www.unfpa.org/public/home/mothers/pid/4385
Lack of bacteriology services leading to no knowledge of bacterial causes of infection, lack of evidence-based antibiotic prescribing, & no understanding of local antibiotic resistance patterns	Establish bacteriological culture at selected district hospitals	
Lack of paediatric nurses, or existing paediatric nurses deployed into administrative roles	Establish a regional training course in highlands for paediatric nurses, with intake particularly from district hospitals	

A programme of training nurses in WHO Hospital Care for Children^7^ which teaches how to use standardized guidelines in everyday clinical practice and includes neonatal resuscitation training, plus programmes of Essential Early Newborn Care and Emergency Obstetric Care^18^,^19^ are resources specifically designed to address such needs at a district hospital. As was discovered, however, despite the existing of these programmes, they are often not active in remote rural district hospitals.

In recent years in PNG, a programme to train doctors in rural medicine through the University of PNG in collaboration with district health services run by churches has begun successfully. Appropriately trained district medical officers can provide leadership, ensuring and maintaining clinical services and other services relevant to the primary role of the district hospital, with support from a provincial paediatrician.

For PNG to achieve MDG health-related targets, gaps in the quality of rural health provision and district hospitals need to be addressed. This study demonstrates that this can be achieved, one quality-improvement step at a time. By setting achievable goals and objectives, identifying key areas of need, training and building capacity of care-providers, and monitoring and evaluating service delivery, sustained progress is possible.

Recent reforms to establish a Provincial Health Authority to bring district hospitals, provincial public health services and provincial hospitals under one administration is a step forward that will hopefully enable ongoing support, communication and exchange of ideas between district medical workers and the provincial hospital paediatricians, and this will be good for the quality of rural health services for children.
